# Cancer Stem Cells in the Thyroid

**DOI:** 10.3389/fendo.2016.00020

**Published:** 2016-02-29

**Authors:** Yuji Nagayama, Mika Shimamura, Norisato Mitsutake

**Affiliations:** ^1^Department of Molecular Medicine, Atomic Bomb Disease Institute, Nagasaki University, Nagasaki, Japan; ^2^Department of Radiation Medical Sciences, Atomic Bomb Disease Institute, Nagasaki University, Nagasaki, Japan

**Keywords:** thyroid cancer, cancer stem cells, thyrosphere, aldehyde dehydrogenase, epithelial–mesenchymal transition

## Abstract

The cancer stem cell (CSC) model posits that CSCs are a small, biologically distinct subpopulation of cancer cells in each tumor that have self-renewal and multi-lineage potential, and are critical for cancer initiation, metastasis, recurrence, and therapy-resistance. Numerous studies have linked CSCs to thyroid biology, but the candidate markers and signal transduction pathways that drive thyroid CSC growth are controversial, the origin(s) of thyroid CSCs remain elusive, and it is unclear whether thyroid CSC biology is consistent with the original hierarchical CSC model or the more recent dynamic CSC model. Here, we critically review the thyroid CSC literature with an emphasis on research that confirmed the presence of thyroid CSCs by *in vitro* sphere formation or *in vivo* tumor formation assays with dispersed cells from thyroid cancer tissues or *bona fide* thyroid cancer cell lines. Future perspectives of thyroid CSC research are also discussed.

## Introduction

The current consensus among cancer biologists is that cancer cellular heterogeneity can be explained in two ways. The first is the stochastic model, in which cancer development is initiated by accumulation of genetic mutations in a single cancer cell, followed by distinct subsequent genetic events in different subpopulations of cells. The second is the cancer stem cell (CSC) model which proposes that there is a small, biologically distinct subpopulation of cancer cells, called CSCs, in each tumor that has self-renewal and multi-lineage potential. Although both models can explain the cellular heterogeneity of cancers, the two models are not mutually exclusive. The CSC model can also help address other poorly understood questions in cancer biology, such as recurrence, metastasis, and therapy-resistance. CSCs were first identified in acute myeloid leukemia in the 1990s, and then in solid cancers in the 2000s (1). In 2007, the first studies on thyroid CSCs and normal thyroid SCs were published (2–4), although there were some earlier papers describing expression of stemness markers in thyroid tissues.

As mentioned above, CSCs can self-renew and differentiate to produce phenotypically diverse cancer cells. In addition, they typically grow *in vitro* as spheres (referred to as thyrospheres in the case of the thyroid), occasionally exhibit chemo/radio-resistance, and share molecular similarities to embryonic and/or adult SCs. In general, the most reliable methods to prove the existence of CSCs experimentally are the *in vivo* tumor formation assay using immunedeficient mice or *in vitro* sphere formation assay using ultra-low attachment plates and serum-free culture. Here, we define thyroid CSCs as fulfilling at least one of these two conditions, although we are aware of the technological concerns surrounding both assays. For an example, *in vivo* tumor assay may just select the cells that can survive in new environments in mice.

In this regard, we critically review the articles published thus far that confirmed the presence of CSCs in thyroid follicular epithelial cell-derived cancers using cell lines or thyroid tumor cells and at least one of the two methods mentioned above (5–19). The studies with cells that were found not to be of thyroid origin are not included in this review (20). It should be noted here that there are some confusions regarding FRO and WRO cells. Schweppe et al. (20) showed that FRO and WRO have wild-type (wt) BRAF and mutant (m) BRAF^V600E^, respectively, while our FRO have mBRAF and the short tandem repeat (STR) profiling data that are similar to those in WRO cells in Schweppe et al., and our WRO have wt BRAF and the unique STR profiling data (8, 21). It is at present unclear which are correct. In our opinion, it is unlikely that follicular thyroid cancer (FTC)-derived WRO cells possess mBRAF. In addition, in Dima et al. (14), cell aggregates were observed within 24 h, implying that they are likely spheroids (22), rather than true thyrospheres.

There is some confusion regarding the use of CSC terminology and the definition of CSCs. Some prefer to avoid the stem cell implications by referring to cells with the ability to initiate tumor growth and serial transplantation in immunocompromised mice as “tumor-initiating cells” (23) or “cancer propagating cells” (24). However, for convenience, we use CSC terminology throughout this review.

## *In Vitro* Thyrosphere and *In Vivo* Tumor Formation Assays

As mentioned above, *in vitro* sphere and *in vivo* tumor formation assays are the most reliable approaches to prove the existence of CSCs. A perfect correlation between these two assays was reported in our studies using eight thyroid cancer cell lines (five anaplastic thyroid cancer (ATC) cell lines (FRO-mBRAF) (21), KTC2, KTC3, ACT1, and 8505C), two papillary thyroid cancer (PTC) cell lines (KTC1 and TPC1), and a FTC cell line (WRO-wt BRAF) (8). Similar correlations were also reported for aldehyde dehydrogenase (ALDH)^+^ cells from 33 out of 34 thyroid cancer samples (6).

The frequency of spherogenic cells has been reported to be variable; 2, 1.2, and 3.5% in dispersed cells from PTC, FTC, and ATC tumors, respectively (6), ~0.1% in dispersed cells from PTC tumors (7), 3.1–9.4% in four ATC cell lines (THJ-11T, -16T, -21T, and -29T) (10), and approximately 0–20% in the ATC cell lines mentioned previously (8). Thyrospheres generated from primary thyroid cancer cells expressed ALDH1A1 and CD44 (6), and some expressed stemness markers, Oct-4, ATP-binding cassette sub-family G member 2 (ABCG2), Sox-2, Nanog, CD133, and CD44 (7, 10, 11). Regarding differentiation markers, PTC-spheres expressed thyroid peroxidase (TPO), thyroglobulin, thyrotropin receptor, thyroid transcription factor 1 (TTF1), and Paired-box 8 (Pax-8) at very low levels (6, 7), while ATC-spheres did not express these markers (6).

*In vivo* tumor formation was achieved by subcutaneous injection of 25,000 cells from PTCs (6) and subcutaneous and orthotopic injections of 500,000 ATC cells mentioned above (10), consistent with the frequency of tumorigenic cells being at least 0.0002–0.004%. However, in our study, as few as 50 cells from ATC cell lines (FRO-mBRAF, KTC3 and ACT1) were tumorigenic when admixed with growth factor-reduced matrigel (8). Enrichment of tumorigenic cells was successful in serial transplantation (10) and seemingly also by using an ALDH marker (6).

## Identification of Thyroid CSCs

Identification of thyroid CSCs has been undertaken using CSC markers that were identified in other solid and hematopoietic malignancies. From a screen of eight thyroid cancer cell lines, we recently reported that there was no common thyroid CSC marker among 11 candidate markers [CD13, 15 (stage-specific embryonic antigen 1, SSEA-1), 24, 44, 44v, 90, 117, 133, 166, and 326 (epithelial cell adhesion molecule, EpCAM), and ALDH] (8). Nevertheless, there have been numerous papers reporting candidate markers for thyroid CSCs. Most studies have been done with a single marker, as summarized below. The usefulness and limitations of each marker have been discussed elsewhere (23, 25, 26).

### Hoechst-Dye Efflux (Side Population)

Side population cells have the ability to exclude the DNA binding dye Hoechst33342 via the ABCG2 drug transporter. It was first reported that small percentages of FRO-mBRAF (0.1%) and WRO-wt BRAF (0.02%) cells, but not TPC1 cells, were SP cells, but no functional analysis was undertaken (2). Similar data (0.43–0.83% positivity rates) were also reported using three ATC cell lines (C643, Hth74, and SW1736), but only SP cells from Hth74 formed thyrospheres (5). Nevertheless, this was the first paper showing sphere formation by thyroid cancer cells. However, this method should be applied carefully because of toxicity of the Hoechst dye and also UV (23, 26).

### Drug Resistance

Long-term culture of Hth74 cells with a low concentration of the anti-cancer drug Doxorubicin led to the establishment of drug-resistant cells (Hth74R), apparently as a result of expansion of pre-existing resistant cells rather than induction of resistant clones (5). The Hth74R cell line contained a much higher percentage of SP cells, and expressed higher levels of a stemness marker (Oct-4) and multidrug-resistance markers [ABCG2, multidrug resistance 1 (MDR1), and multidrug resistance-associated protein 1 (MRP1)] compared to the parental Hth74 cells (5). However, comparison of the efficacy of thyrosphere formation by parental Hth74 and drug-resistant Hth74R cells was not addressed. Although SP cells in the parental Hth74 cell line were shown to be spherogenic as mentioned above, an increase in number of SP cells does not necessarily mean an increase in CSCs. Thus, this study may represent the establishment of drug-resistant cell lines expressing higher levels of ABC transporters.

Expression of drug-resistance genes in CSCs may help explain the chemo-resistance of CSCs. However, CSCs usually proliferate slowly (even slower than non-CSCs) and are occasionally in the G0/G1 phase (i.e., dormant cells), which may contribute to the resistance to anti-cancer treatments, because quiescent cells are usually more drug-resistant than proliferative cells.

### Aldehyde Dehydrogenase

Aldehyde dehydrogenase plays roles in a variety of biological activities, including oxidation of toxic cellular aldehyde to carboxylic acids, biosynthesis of retinoic acid that binds to the heterodimeric transcription factor composed of retinoic acid receptor and retinoid x receptor, resistance to chemotherapeutic treatments [see reviews in Ref. (27–29) for more details]. As an example, ALDH1A3 was reported to be involved in the resistance of K1 thyroid cancer cells to a FAK inhibitor (Y15) (30). By contrast, all-trans-retinoic acids, a product of ALDH catalysis, induced re-differentiation of thyroid cancer cells, consistent with ALDH as a potential therapeutic target (31).

In our opinion, the most elegant study of thyroid CSCs so far published is by Todaro et al. (6). They showed that thyrospheres can be derived from dispersed cells from PTC, FTC, and ATC tumors. These spheres expressed ALDH1A1, CD44 and stemness markers (Oct3/4 and Nanog), and ALDH^+^ cells were more spherogenic and tumorigenic than ALDH^−^ cells. Thus, subcutaneous injection of 5,000 ALDH^+^, but not 25,000 ALDH^−^ cells, produced tumors in immunocompromised mice, and as few as 100 ALDH^+^ cells were also tumorigenic when injected orthotopically. This is the lowest cell number required for *in vivo* tumor formation in thyroid CSC studies published to date, except our study using matrigel (see above) (8). Reeb et al. also reported successful tumor formation by orthotopic injection of 100 thyrosphere cells from ATC cell lines, THJ-11T and THJ-16T (18).

In our studies with thyroid cancer cell lines (8), ALDH positivity rates ranged from 6 to 85% in five ATC cell lines, 0–36% in two PTC cell lines, and 9% in one FTC cell line. Four ATC cell lines (FRO-mBRAF, ACT1 and KTC3, 8505C) developed thyrospheres *in vitro* and tumors *in vivo* in nude mice. Four other PTC, FTC, or ATC cell lines that were tested did not form thyrospheres *in vitro*, or tumors *in vivo*. ALDH^+^ cells from the FRO-mBRAF, ACT1 and KTC3 (but not 8505C) cell lines were more spherogenic than ALDH^−^ cells. Of interest, ALDH expression was flexible, that is, ALDH^+^ cells could be derived from both ALDH^+^ and ALDH^−^ cells. Furthermore, ALDH^−^ cells were comprised of CD326 (EpCAM)^+^ and CD326^−^ cells, and the spherogenic activity of the former was higher than the latter, and comparable to that of ALDH^+^ FRO-mBRAF cells.

In the study by Todaro et al., ALDH1A1 was expressed in a small number of thyroid cancer cells, and the percentages of ALDHA1 expressing cells (3% in FTC, 7% in PTC, and 16% in ATC) were positively correlated with aggressiveness (6). However, other studies, including ours (32, 33), reported much higher positivity rates for ALDH1A1 expression in normal and PTC tissues by immunohistochemistry using the same antibody. Although the reason(s) for these differences is/are at present unclear, and the latter data do not immediately dismiss the significance of ALDH as a CSC marker, Deng et al. concluded that “…ALDH should not be used as a CSC marker in tissue types that normally express a high level of ALDH1” (29, 33).

It should be noted here that of the 19 ALDH isozymes, ALDHA1 is the most predominantly expressed in both normal and cancerous thyroid tissues (32). However, ALDH1A3 is the most highly expressed isozyme in the K1 thyroid cancer cell line mentioned above (30).

### CD133

CD133, also called prominin-1, is a five-trans membrane domain glycoprotein with unknown function and serves as a marker of stem cells in many normal and cancerous cells. Tseng et al. isolated CD133^+^ cells from ATC tumors and the ATC cell lines, BHT-101, CAL-62, and 8505C. The CD133^+^ cells expressed stemness genes (Oct-4, Sox-2, and Nanog) and drug-resistance genes (ABCG2, MDR1, and MRP), were chemo-resistant, and formed thyrospheres *in vitro* and tumors *in vivo* (9). However, the positivity rates of CD133 were not described. Signal transducer and activation of transcription 3 (STAT3) signaling was activated in CD133^+^ cells compared to CD133^−^ cells, and inhibition of STAT3 signaling with a JAK–STAT inhibitor (cucurbitacin I) decreased CD133^+^ cell proliferation in adherent culture, and sphere formation in suspension culture. However, the effect of cucurbitacin I was only specifically evaluated in the CD133^+^ fraction of cells in some experiments, and when studied in both fractions in other experiments, its effect on some cell functions did not appear to differ.

Furthermore, and more generally, the significance of CD133 as a CSC marker is controversial. When considering thyroid cancer specifically, CD133 mRNA was expressed in spheres from dispersed thyroid cancer cells (7, 13). CD133 protein was expressed in a FRO cell line (BRAF status unknown) but not in a TPC cell line (34), and not in eight cell lines, including FRO-mBRAF (8) and cancer-derived thyrospheres/tumors (6, 10).

Furthermore, CD133 data should be interpreted with caution because the antibodies against CD133 used in all the studies mentioned above is the clone AC133 (or CD133/1), which was previously reported to recognize only glycosylated versions of CD133 rather than unmodified CD133 protein isoforms (35).

### CD44 and CD24

Ahn et al. focused their attention on CD44 and CD24, which are CSC markers for some cancers, including breast (11). Using cancer cell lines (the original TPC1 cell line and its derivatives), they found higher numbers of CD44^+/^CD24^−^ cells in the more aggressive cell lines (positivity rates being 86% in highly tumorigenic TPC-1Mice cells, >73% in moderately tumorigenic TPC-1SC2 cells, >21% in parental, poorly tumorigenic TPC-1 cells). Subsequently, using dispersed cells from thyroid cancers, they determined that 4–70% cells were CD44^+^CD24^−^, the cells formed spheres, CD44^+^CD24^−^ but not CD44^+^CD24^+^ cells from these spheres were spherogenic, and the cells derived from thyrospheres (at least 1 × 10^4^) formed tumors following orthotopic injection.

In our study, CD24 was not expressed on TPC1 cells, but all WRO-wt BRAF, ACT1, and 8505C cells expressed CD24. CD44 was expressed on 100% of cells in all the cell lines examined, and CD44v8-10, a CD44 variant, including exons 8–10, was expressed in two ATC cell lines (FRO-mBRAF and ACT1), both of which were spherogenic/tumorigenic (8). CD44v8-10 contributes to protection from reactive oxygen species-induced cellular stress, a typical CSC characteristic, by producing reduced glutathione, the primary intracellular antioxidant (36).

### Stage-Specific Embryonic Antigen-1 (or CD15)

Stage-specific embryonic antigen-1 was expressed at variable levels in all of the cell lines examined (ML1, FTC236, FTC238, T238, SW1736, and TPC1; 3–9%) in one study (16), but only in one (FRO-mBRAF) of eight cell lines in another report (8). In the former, SSEA-1^+^ cells expressed stemness and epithelial–mesenchymal transition (EMT) (see below) markers, and 10^4^ SSEA-1^+^ cells produced subcutaneous tumors, whereas SSEA-1^−^ cells did not. Of interest, SSEA expression exhibited plasticity, suggesting that SSEA^+^ and SSEA^−^ cells were in a dynamic equilibrium (see below).

## Induction of CSCs from Bulk Cancer Cell Preparations

Epithelial–mesenchymal transition was originally recognized to be a vital process for morphogenesis in embryonic development, and was recently implicated in invasion/metastasis of malignancies and the biology of CSCs (37). Indeed, in our studies (8, 12), although the cells with epithelial phenotype (ACT1) and those with mesenchymal phenotype (FRO-mBRAF and KTC3) were both capable of forming thyrospheres, the spherogenicity was higher in the latter.

A correlation between forced induction of EMT status and stemness was reported in two articles, which used the transcription factors SNAIL (12), and hypoxia-inducible factor (HIF)-1α (15), as EMT-inducers in epithelial type ACT1 and FTC133 cells, respectively. EMT phenotypes, such as decreased expression of epithelial markers (e.g., E-cadherin), increased expression of mesenchymal markers (e.g., vimentin), and spindle-shaped appearance, were induced in both studies. In one study, EMT induced by overexpression of SNAIL in ACT1 led to an increase in thyrosphere forming ability in a small fraction of ALDH^−^ cells, indicating a link between EMT and CSCs only in ALDH^−^ cells (12). In the other report, EMT induction by HIFα was accompanied by high invasiveness and increased numbers of sphere-forming SP cells expressing the stemness gene Oct-4, and the drug-resistance gene ABCG2 (15).

However, in the latter, the morphologies of the parental and transfected cells (cobblestone- and spindle-shaped appearances, respectively) and those of SP and non-SP cells isolated from transfected cells (cobblestone- and spindle-shaped appearances, respectively) in adherent culture could not be reconciled. Similar data were also presented in a previous paper by the same authors (5). Furthermore, because an EMT phenotype is generally observed at the invasive fronts of thyroid cancer tissues (38, 39), while HIF-1α is usually expressed in cells in hypoxic, central regions of cancers, the significance of HIFα-induction of EMT in thyroid cancers is unclear.

Very recently, the significance of EMT in invasion and metastasis was challenged, and instead implicated in the development of chemo-resistance (40).

## Comparison of Normal Thyroid SCs and Thyroid CSCs

Thyrospheres have been generated from normal thyroid cells as well as those from Graves’ and nodular goiter tissues (4, 13, 41). SP cells purified from nodular goiters (Oct-4^+^ and thyroid differentiation markers^−^) did not proliferate independently, while the bulk of dispersed cells from the same tissues, which was contaminated with endodermal cells, did produce thyrospheres (4), suggesting that co-existing endodermal cells may be important as niche cells. By contrast, as mentioned above, some cancer cell lines and cancer cells can form spheres independently.

A comparison of normal thyroid SCs and thyroid CSCs was performed in detail by Giani et al. (13). PTC-CSCs exhibited the higher clonogenic potential, forming larger, more irregular spheres, as compared to normal SCs. PTC–CSCs also expressed higher levels of stemness markers (Oct-4, Sox-2, and ABCG2) and an EMT marker (vimentin), lower levels of some differentiation markers (Pax-8 and TTF1), and showed poorer differentiation efficiency than normal SCs. Several genes and gene families were differentially expressed between normal SCs and CSCs, including Wnt pathway, whose expression levels were higher in CCSs than normal SCs.

## Signaling Pathways Controling CSC Properties

The successful development of CSC-targeted therapies may be achieved by targeting CSC-specific cell surface markers or signal transduction pathways. In this regard, it is important to delineate the signaling pathways controlling CSC initiation and growth. The following three different intracellular signal transduction pathways are important mediators of thyroid CSC biology.

### The Insulin-Like Growth Factor-I (IGF-I) Pathway

Papillary thyroid cancer-spheres expressed insulin-like growth factor (IGF)-I/II and IGF-IR, and stimulation of this signaling pathway increased the number and size of spheres (7).

### The Sonic Hedgehog Signaling Pathway

The sonic hedgehog (Shh) pathway is activated in some ATC cell lines (SW1736, BCPAP, and KAT-18), and pathway inhibitors and shRNA-mediated suppression of Shh signaling molecules inhibited ALDH activity and thyrosphere formation (17). The importance of Shh was also demonstrated by increased sphere formation by overexpression of the Shh pathway effector molecule, Gli1. However, a caveat of these experiments is that it was unclear whether the effects of these inhibitors and shRNA were CSC-specific.

### The STAT3 Signaling Cascade

This pathway was activated in ATC-CD133^+^ cells, and the suppressive effect of a JAK–STAT inhibitor cucurbitacin I on CSC characteristics was shown (9).

### Others

The therapeutic effects of targeting other stem cell markers, including Sox-2 or CD44 were reported using bulk cancer cells, although the CSC fractions were not studied in isolation (42, 43).

## Cell-of-Origin of CSCs and Hierarchical Versus Dynamic Models

The cellular origins of thyroid CSCs are unknown, and it remains to be determined whether they are derived from normal SCs, progenitor cells or mature thyroid cells. The thyroid fetal cell carcinogenesis hypothesis (44) was proposed over a decade ago, but has not yet been substantiated. Experiments with the thyroid cancer mouse model (BRAF-V600E^f/wt^; TPO-Cre) demonstrated that cancer cells developed from TPO-expressing (i.e., at least partially differentiated) thyroid cells and showed EMT characteristics, consistent with the derivation of CSCs from differentiated thyroid cells via EMT, rather than from normal SCs (45). However, these data should be interpreted with caution because human thyroid sphere cells and mouse thyroid SP cells express low levels of TPO (3, 13).

The original and somewhat rigid hierarchical CSC model proposed that CSCs lie at the apex of the hierarchical organization of cancer tissues, and have abilities to self-renew and unidirectionally differentiate to progenitor and mature cancer cells. However, this model has recently been challenged. In the dynamic CSC model, CSC phenotypes are more flexible, and cells interconvert between CSCs and non-CSCs spontaneously, or in response to particular conditions (46, 47). A good example is EMT induction of CSCs as mentioned above. Furthermore, it was demonstrated that ALDH^+^ CSCs (8) and SSEA-1^+^ CSCs (16) exhibited plasticity by spontaneously transitioning from one state to the other in dynamic equilibrium. Thus, the data so far obtained for thyroid cancers are more consistent with the dynamic CSC model.

The dynamics of CSC properties have been demonstrated in several other cancers. Some showed spontaneous equilibrium between CSCs and non-CSCs (48, 49), and others showed induction of CSC characteristics by particular signals (e.g., EMT and IL-6) (37, 50).

## Discussion

In this review, we have described the significant progress that has been made in addressing the biology of thyroid CSCs. However, numerous questions remain unanswered and new issues have arisen as the field has expanded. We discuss some of these issues below.

Numerous CSC markers have been identified to date (SP, drug resistance, expression of ALDH, CD133, CD24, and SSEA 1). With respect to these markers, thyroid cancers are similar to other malignancies in terms of expression of CD44^+^CD24^−^, ALDH^+^, and CD133^+^ (breast cancers), CD133^+^ and ALDH^+^ (colon cancers), and CD44^high^ESA^low^-EMT and CD44^high^ESA^high^-EMT in oral squamous cell carcinomas (see Ref. (8) for more details). However, for thyroid cancers (and most other tumors), it is unclear whether these multiple markers represent discrete or overlapping CSC subpopulations or whether there are other as yet unidentified definitive CSC markers. It also remains to be clarified whether different tumors express distinct and characteristic CSC markers, or individual cancers contain multiple different types of CSCs.

It is important to acknowledge that the data summarized in this review may at least in part reflect experimental artifacts relating to experimental conditions and/or cell types being studied, and technical variability across different laboratories. For example, one paper reported thyrosphere formation only from ATC cell lines, not differentiated thyroid cancer (DTC) cell lines (8), while others described thyrospheres from cell lines or dispersed cells from DTCs (6, 11, 13). Also ALDH1A1 positivity rates in normal/cancerous thyroid tissues/cells and expression levels of CD133, SSEA-1, and CD24 in thyroid cancer cells varied among different studies. Furthermore, improvements in the efficiency of CSC isolation are clearly necessary because even after CSC enrichment using a particular marker, only a small fraction of the marker^+^ cells are spherogenic/tumorigenic, consistent with only partial purification of CSCs.

Similarly, multiple signaling pathways, such as IGF-1, STAT3, and Shh, play a role in regulating thyroid CSC growth. STAT3 and Shh signaling are linked to the biology of CD133 and ALDH, respectively, but the relative importance of the crosstalk between these pathways remains enigmatic. Furthermore, although several reports have pointed to the potential of some drugs and shRNA for CSC therapy, the specificities and effectiveness of these agents have not been fully evaluated. Importantly, the relative effects of each agent on CSCs and non-CSCs (or at least the bulk of cells) should be carefully addressed. In our opinion, “CSC-targeted therapies” should show specific killing of CSCs, and not non-CSCs, including, in particular, normal SCs. We believe that a therapeutic agent that kills both CSCs and non-CSCs is not CSC-targeted.

Finally, whether thyroid CSC biology reflects the original, hierarchical CSC model or the more flexible, dynamic model is another important issue. The data acquired so far (albeit from only two cell lines) indicate that thyroid CSC biology is more consistent with the dynamic model. In this regard, studies on genetic/epigenetic/environmental signaling networks regulating CSC plasticity will be intriguing. In addition, plasticity of CSC phenotype may challenge the idea that the study of CSCs is pivotal for the development of novel treatment modalities for thyroid cancer. CSC-targeted therapy may not be curative because of spontaneous reprograming from non-CSCs to CSCs. Overall, although the potential of CSC-targeted therapy for treating thyroid cancer remains tantalizing, further studies are necessary to comprehensively understand thyroid CSC biology and ultimately achieve the goal of curing thyroid cancer patients by CSC-targeted therapy.

## Author Contributions

All the authors (YN, MS, and NM) equally contributed to write this review article.

## Conflict of Interest Statement

The authors declare that the research was conducted in the absence of any commercial or financial relationships that could be construed as a potential conflict of interest.
